# The association between the degree of nausea in pregnancy and subsequent posttraumatic stress

**DOI:** 10.1007/s00737-018-0909-z

**Published:** 2018-09-17

**Authors:** Helena Kames Kjeldgaard, Åse Vikanes, Jūratė Šaltytė Benth, Carolin Junge, Susan Garthus-Niegel, Malin Eberhard-Gran

**Affiliations:** 1 Health Services Research Unit, Akershus University Hospital, Lørenskog, Norway; 2 Institute of Clinical Medicine, Campus Ahus, University of Oslo, Lørenskog, Norway; 3 The Intervention Centre, Oslo University Hospital, Oslo, Norway; 4 Department of Child Health, Norwegian Institute of Public Health, Oslo, Norway; 5 Institute and Policlinic of Occupational and Social Medicine, Faculty of Medicine, Technische Universität Dresden, Dresden, Germany; 6 Department of Psychotherapy and Psychosomatic Medicine, Faculty of Medicine, Technische Universität Dresden, Dresden, Germany

**Keywords:** Hyperemesis gravidarum, Nausea and vomiting, Pregnancy, Mental health, Posttraumatic stress disorder

## Abstract

Hyperemesis gravidarum (HG) is a pregnancy condition characterised by severe nausea and vomiting during early pregnancy. The experience of HG is for many women a traumatic event. Few studies have investigated a possible association between HG and birth-related posttraumatic stress. The objective of the current study was to assess whether HG increases the risk of birth-related posttraumatic stress symptoms (PTSS). This was a population-based pregnancy cohort study using data from the Akershus Birth Cohort Study (ABC study). A linear mixed model was used to estimate the association between the degree of nausea (no nausea (*n* = 574), mild nausea (*n* = 813), severe nausea (*n* = 522) and HG (hospitalised due to nausea, *n* = 20)) and PTSS score at 8 weeks and 2 years after birth. At 8 weeks postpartum, women with HG had higher PTSS scores compared to women with no nausea (*p* = 0.008), women with mild nausea (*p* = 0.019) and women with severe nausea (*p* = 0.027). After 2 years, women with HG had higher PTSS scores compared to women with no nausea (*p* = 0.038). Women with HG had higher PTSS scores following childbirth compared to women with less pronounced symptoms or no nausea at all. After 2 years, women with HG still had higher PTSS scores compared to women with no nausea. Although the overall differences in PTSS scores were small, the results may still be of clinical relevance.

**Electronic supplementary material:**

The online version of this article (10.1007/s00737-018-0909-z) contains supplementary material, which is available to authorized users.

## Introduction

Nausea and vomiting in pregnancy affects up to 80% of all pregnancies, predominantly during the first trimester (Gadsby et al. 1993), and is a common cause of sick leave (Dorheim et al. 2013). An estimated 0.3 to 2% of women develop a very severe form, known as hyperemesis gravidarum (HG), which may include metabolic disturbances and nutritional deficiencies (Eliakim et al. 2000; World Health Organization 2004), and therefore lead to hospitalisation (Gazmararian et al. 2002).

The exact cause of HG is not known. However, one of the main hypotheses of today involves the rise of the pregnancy hormone, human chorionic gonadotrophin (hCG), which coincides with the occurrence of HG. Several conditions associated with increased hCG, such as molar pregnancy and multiple gestation, are associated with increased risk of developing HG (Grooten et al. 2015). Another hypothesis involves genetics as HG is inherited along the maternal line (Vikanes et al. 2010a). Moreover, recent, genetic research has identified genes that may increase the risk of HG (Fejzo et al. 2017, 2018). HG has, however, a long history of stigmatisation, being described as the result of hysterical personality traits and psychological disturbances (Fairweather 1968). Despite recent research, women with therapy-resistant HG nevertheless continue to be examined for psychiatric disorders (Kim et al. 2009). A growing body of research has shown an association between HG and maternal depression and anxiety during pregnancy (Mitchell-Jones et al. 2017); however, the causal direction of this association remains elusive (Aksoy et al. 2015; Fell et al. 2006; Kjeldgaard et al. 2017a; Pirimoglu et al. 2010; Seng et al. 2007).

The few studies that have researched psychological distress following pregnancy suggest that HG increases the risk of postpartum depression (Kjeldgaard et al. 2017b) and stress reactions (Christodoulou-Smith et al. 2011; Meltzer-Brody et al. 2017), in particular, for those experiencing prolonged or extreme symptoms (Fejzo et al. 2009; Kjeldgaard et al. 2017b; Mullin et al. 2012). It may therefore be hypothesised that HG could influence the whole pregnancy and birth experience in such a way that it causes posttraumatic stress symptoms (PTSS) following childbirth. A lack of a holistic approach, including psychosocial as well as physical care by midwives, nurses and medical doctors, may worsen the psychological distress of women with HG (Poursharif et al. 2008; Power et al. 2010), further increasing the risk of PTSS (Ayers et al. 2016; Grekin and O'Hara 2014; Reed et al. 2017).

The aim of the present study was to estimate the association between the degree of nausea in pregnancy and birth-related PTSS and whether PTSS change from 8 weeks to 2 years after childbirth.

## Methods

### Study population

**Fig. 1:**
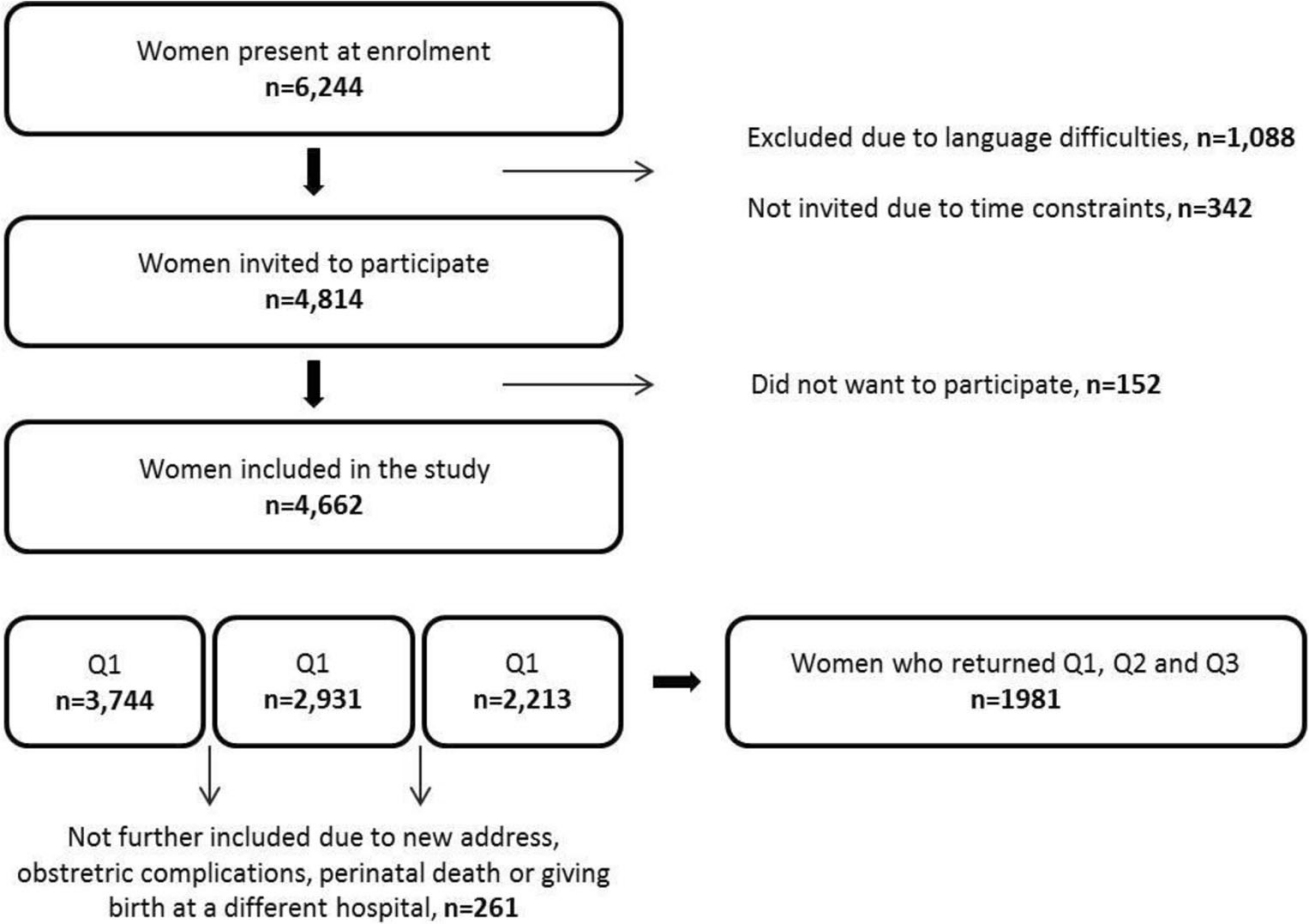
Flowchart showing the enrolment into the Akershus Birth Cohort study, Norway, 2008–2012

Data were derived from the Norwegian Akershus Birth Cohort (ABC), a large population-based prospective cohort study. Between November 2008 and April 2010, women were recruited at the routine foetal ultrasound examination in pregnancy week 17 (Garthus-Niegel et al. 2014, 2017). There were no exclusion criteria other than inability to complete a questionnaire in Norwegian. Of the 4814 women invited to participate, 4662 women were included in the study (96.8%). Data were collected by self-administered questionnaires in pregnancy weeks 17 (Q1) and 32 (Q2), and 8 weeks (Q3) and 2 years (Q4) after delivery. Q3 was administered 8 weeks following childbirth to ensure that maternity blues (Pop et al. 2015) would not interfere with the assessment of postpartum psychiatric disorders. Q4 was administered 2 years after birth, as the main focus of this questionnaire was to assess mental health in relation to child development. Additional information was obtained by linkage to the electronic birth records. The birth records are completed by the hospital staff and contain socio-demographic and medical information about the mother, child, pregnancy and birth. Some women dropped out of the study due to moving or severe obstetric complications. Figure 1 displays a flow chart of the recruitment and retention for the first three questionnaires, which comprised our baseline study sample. Note that the sample sizes may deviate somewhat from previous publications due to small changes in the latest quality-assured data files released for research.

**Fig. 2:**
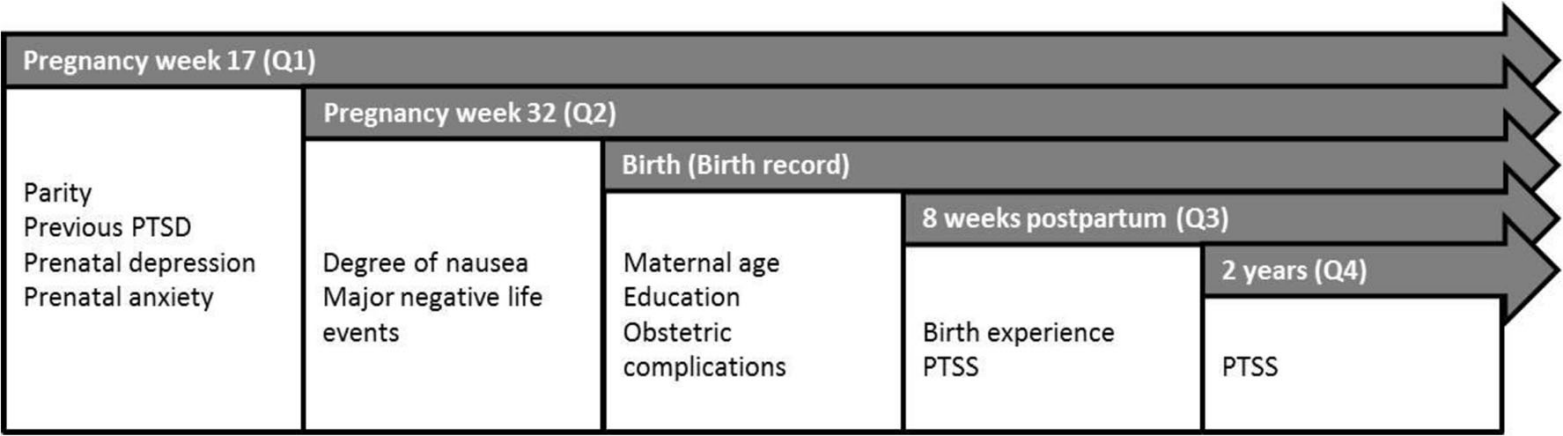
An overview of the relevant variables measured in the questionnaires and birth record, Akershus Birth Cohort study, Norway, 2008–2012

We included all women who participated in at least the first three questionnaires (*n* = 1981). Women with multiple pregnancies were excluded (*n* = 36). The final study sample consisted of 1945 women. An overview of time points for data collection of relevant maternal characteristics is presented in Fig. 2.

All women gave written informed consent. The study was approved by the Regional Committee for Ethics in Medical Research in Norway (approval number S-08013a)

### Key variables

The main predictor was the degree of nausea, reported in Q2. The women reported whether they had experienced no, mild or severe nausea, and whether they had been on sick leave or hospitalised due to nausea. We categorised the degree of nausea into four groups: no nausea, mild nausea, severe nausea (including sick leave) and HG (hospitalised due to nausea).

The main outcome was PTSS, measured by the Impact of Event Scale (IES) (Horowitz et al. 1979) in Q3 and Q4. The questions were asked in relation to the birth of the index pregnancy. The IES is a self-rating scale that measures symptoms of intrusion (seven items) and avoidance (eight items). The scale has four response categories (not at all, rarely, sometimes and often), with higher scores reflecting a higher degree of posttraumatic stress. The scale has been validated in postpartum women (Olde et al. 2006), with scores over 19 reflecting clinically significant distress, and scores above 34 indicating probable presence of PTSD (Neal et al. 1994).

### Mental health measurements

Symptoms of previous general posttraumatic stress disorder (PTSD) were measured in Q1. The women reported whether they at any time in their lives had been involved in or had experienced a dramatic and terrifying event. If they had, they reported whether they had suffered from any of eight potential symptoms related to that event during the last month. The symptoms were based on the Mini-International Neuropsychiatric Interview (M.I.N.I.), which is designed for epidemiological studies (Sheehan et al. 1998). The measured symptoms were (1) re-experienced the event (e.g., in dreams, nightmares, intense memories or flashbacks), (2) avoided thinking or talking about the event, (3) had problems remembering the event, (4) felt distant, (5) had been considerably disturbed by the event in my work and in social activities, (6) had problems sleeping, (7) had problems concentrating or (8) had been nervous. A score of 1 was given if the symptom was present, resulting in a sumscore ranging from 0 (no symptoms) to 8 (maximum number of symptoms).

Prenatal depression was measured by the Edinburgh Postnatal Depression Scale (EPDS) (Cox et al. 1987) in Q1. The EPDS is a 10-item self-rating scale designed to identify postnatal depression but has also been validated in pregnancy (Cox et al. 1996; Eberhard-Gran et al. 2001; Murray and Cox 1990). Each item is scored from 0 to 3 with higher scores reflecting higher levels of depression. The total score ranges from 0 to 30. Prenatal depression was dichotomised as “yes” and “no” with presence of depression defined as a score > 12.

Anxiety was measured using 10 items from The Hopkins Symptom Check List (SCL-25) (Hesbacher et al. 1980) in Q1. The SCL-25 is a widely used self-rating scale, and the first 10 items comprise the anxiety score (SCL-A). Each item is scored from 0 to 3 with higher scores reflecting higher levels of anxiety. Each item ranges from “not at all” (score 1) to “extremely” (score 4), and the sumscore ranges from 10 to 40. Presence of anxiety was defined as a score ≥ 18.

Subjective birth experience was measured using a numeric rating scale (NRS) 8 weeks after birth. The NRS was based on the question “What was your overall experience of the birth?” The answers were scored from 0 (“very good”) to 10 (“extremely bad”). Negative birth experience was defined as an NRS score ≥ 9.

### Socio-demographic and reproductive factors

Parity was obtained from Q1 and dichotomised into primiparity and multiparity. Maternal age and education were obtained from the birth record. Level of education was dichotomised into ≤ 12 years and > 12 years.

Using information on birth complications registered in the birth record, we constructed an index of obstetric complications, which have previously been associated with HG and/or postpartum PTSD, and thus may influence the association between nausea and PTSS. Each potential complication was treated as a dichotomous variable, depending on whether or not it had occurred. As few women had more than one of the included obstetric complications, two categories were created; “no complications” and “1 complication or more”. The complications were the following: (1) preterm birth (< 37 weeks), (2) placental abruption, (3) pre-eclampsia, (4) small for gestational age and (5) low 10-min Apgar score (Bolin et al. 2013; Dodds et al. 2006; Fiaschi et al. 2018; Meltzer-Brody et al. 2017).

Major negative life events during the last 12 months were measured using ten items in Q2. These items were selected from established life event scales (Coddington 1972; Swearingen and Cohen 1985). The life events included were (1) being separated or divorced, (2) serious problems in the marriage or cohabiting relationship (3) problems or conflicts with family members, friends or neighbours, (4) problems at work or in school, (5) economic problems, (6) serious illness or injury, (7) serious illness or injury in the close family, (8) traffic accident, fire or theft, (9) loss of a closely related person and (10) other difficulties. As few women experienced more than one event, major negative life events were dichotomised as “yes” with one or more negative life events present or “no”.

### Statistics

Data were described as means and standard deviations (SD) or frequencies and percentages. Missing values on the psychometric scales were imputed if the number of missing items was ≤ 20% per case. The association between PTSS scores assessed at 8 weeks and 2 years after giving birth and the degree of nausea was assessed by a linear mixed model with random intercepts for women, accounting for intra-women correlations. Fixed effects for time point (8 weeks or 2 years), degree of nausea and the interaction between those two were entered. Further, to assess whether possible confounders (parity, maternal age, education, obstetric complications, previous PTSD, prenatal depression, prenatal anxiety, major negative life events) were associated with changes in PTSS over time, the multiple linear mixed model with fixed effects for each characteristic and the interaction between the characteristic and time point were estimated. In order to explore, whether the differences in birth-related PTSS scores were explained by negative birth experience, we performed two models adjusting for co-variates: one multiple model with no adjustment for negative birth experience and one multiple model adjusting for negative birth experience.

Linear mixed modelling handles unbalanced data by including all available information, also from drop-outs.

SAS version 14 was used for data analysis. The results with *p* values below 0.05 were considered statistically significant.

#### Data availability

The dataset analysed in the current study is not publicly available due to data privacy restrictions and ethical restrictions established by the Norwegian Regional Committee for Ethics in Medical Research. Data are however available through application to the ABC study. All inquiries about access to data should be sent to the ABC steering group, attention: Nina.Vislokken.Odegard@ahus.no. All requests to access personal data will be handled in accordance with the procedures by the Ethics Committee.

## Results

A total of 1945 women were included in the study. The mean age was 31.3 years (range 18.8–45.5 years; SD 4.6 years), and 50% were primiparous. Roughly 70% of the women reported nausea; 42% had mild nausea, nearly 27% had severe nausea and 1% had HG. A total of 72 (3.7%) and 35 (1.8%) women reported birth-related PTSD (PTSS score > 34) 8 weeks and 2 years after birth, respectively. Characteristics of the sample are presented in Table 1.

**Table 1:** Demographic characteristics and other factors of 1945 women 8 weeks and 2 years after birth, Akershus Birth Cohort study, Norway, 2008–2012

Time Co-variate	8 weeks	2 years
	(*n* = 1930)	(*n* = 1305)
	*n* (%)	*n* (%)
Nausea		
None	574 (29.8)	396 (30.4)
Mild	813 (42.1)	540 (41.4)
Severe	522 (27.1)	357 (27.4)
HG	20 (1.0)	11 (0.8)
Parity		
Primipara	964 (49.9)	682 (52.3)
Multipara	966 (50.1)	623 (47.7)
Education		
≤ 12 years	595 (30.9)	349 (27.7)
> 12 years	1268 (68.1)	909 (72.3)
Obstetric complications		
None	1558 (84.2)	1046 (83.0)
1 or more	291 (15.8)	215 (17.0)
Negative birth experience		
No	1827 (95.4)	1232 (95.1)
Yes	89 (4.6)	63 (4.9)
Prenatal depression		
No	1806 (93.8)	1231 (94.4)
Yes	120 (6.2)	73 (5.6)
Prenatal anxiety		
No	1761 (92.3)	1201 (92.8)
Yes	147 (7.7)	93 (7.2)
Major negative life events		
None	1053 (54.6)	723 (55.4)
1 or more	877 (45.4)	582 (44.6)
	Mean (SD)	Mean (SD)
Maternal age Previous PTSD	31.3 (4.6) 0.22 (0.71)	31.6 (4.5) 0.19 (0.66)

According to linear mixed model, there were statistically significant differences in PTSS scores according to the degree of nausea. As compared to women without nausea, at 8 weeks after birth, women with severe nausea (*p* = 0.002) and with HG (0.006) had statistically significantly higher PTSS scores. Women with HG also had statistically significantly higher PTSS scores compared to women with mild nausea (*p* = 0.023). Two years after birth, only women with severe nausea had statistically significantly higher PTSS scores compared to the scores of women without nausea (*p* = 0.035).

When adjusted for co-variates (parity, maternal age, education, obstetric complications, previous PTSD, prenatal depression, prenatal anxiety and major negative life events), the results slightly changed. In the model not adjusting for negative birth experience, there were no longer statistically significant differences in PTSS scores between women with mild nausea and women with HG (*p* = 0.054) 8 weeks postpartum.

**Fig. 3:**
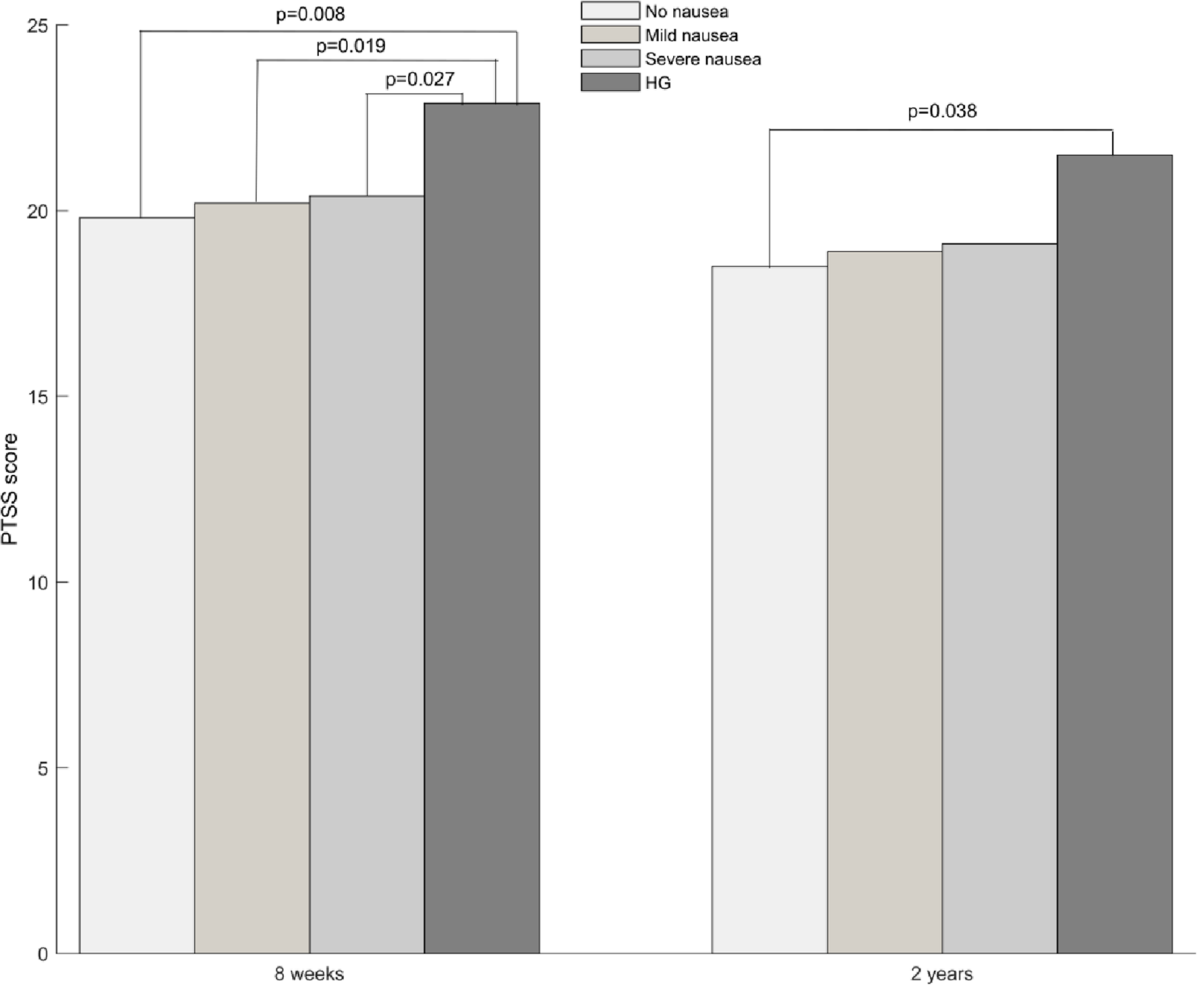
Mean PTSS scores at 8 weeks and 2 years after birth among different nausea groups, Akershus Birth Cohort study, Norway, 2008–2012

The results changed in the model adjusting for negative birth experience. Higher PTSS was significantly associated with negative birth experience (*p* < 0.001) at both time points. Eight weeks after giving birth, women with HG had higher PTSS scores compared with women without nausea (*p* = 0.008), women with mild nausea (*p* = 0.019) and women with severe nausea (*p* = 0.027). After 2 years, women with HG only had higher PTSS scores compared with women without nausea (*p* = 0.038). The main results are listed in Table 2 and presented graphically in Fig. 3.

**Table 2:** Unadjusted and adjusted mean PTSS scores, of main variables, with 95% CI and *p* values at 8 weeks (*n* = 1749) and 2 years (*n* = 1193) after birth, Akershus Birth Cohort study, Norway, 2008–2012. Cases with at least one missing value on co-variates were excluded

Variable	Unadjusted		Adjusted, without negative birth experience		Adjusted, with negative birth experience	
	8 weeks	2 years	8 weeks	2 years	8 weeks	2 years
Nausea						
No – ref.	19.6 (19.1; 20.1)	18.6 (18.1; 19.1)	19.8 (18.0; 21.6)	18.5 (16.4; 20.7)	19.8 (18.1; 21.6)	18.5 (16.4; 20.5)
Mild	20.2 (19.8; 20.6)	19.1 (18.6; 19.5)	20.4 (18.6; 22.1)	19.1 (17.0; 21.1)	20.2 (18.5; 21.9)	18.9 (16.9; 20.9)
Severe	20.7 (20.2; 21.1)	19.4 (18.9; 20.0)	20.6 (18.8; 22.4)	19.3 (17.2; 21.3)	20.4 (18.7; 22.1)	19.1 (17.1; 21.0)
HG	23.1 (20.6; 25.5)	21.5 (18.4; 24.6)	22.6 (19.8; 25.5)	21.3 (17.8; 24.8)	22.9 (20.2; 25.6)	21.5 (18.1; 25.0)
No vs. mild	0.057	0.180	0.077	0.107	0.184	0.177
No vs. severe	*0.002*	*0.035*	*0.018*	*0.047*	0.099	0.115
No vs. HG	*0.006*	0.068	*0.019*	0.068	*0.008*	*0.038*
Mild vs. severe	0.158	0.335	0.406	0.567	0.623	0.702
Mild vs. HG	*0.023*	0.126	0.054	0.141	*0.019*	0.075
Severe vs. HG	0.057	0.193	0.087	0.181	*0.027*	0.092

Adjusted for maternal age, parity, education, obstetric complications, previous PTSD, prenatal depression, prenatal anxiety and major negative life events. Numbers depicted in italics represent statistically significant values at significance level 5%.

Regarding co-variates, the following were statistically significantly associated with PTSS at both time points in the unadjusted and the two adjusted model: previous PTSD, parity, prenatal depression and negative life events. Obstetric complications were not associated with PTSS, while level of education (at 8 weeks postpartum) and maternal age (at 8 weeks and 2 years after giving birth) were associated with PTSS in the unadjusted model but not in the adjusted models. Prenatal anxiety was statistically significant associated with PTSS in the unadjusted model but only at 8 weeks in the adjusted models. The results for all included variables are presented in the supplementary table.

## Discussion

The main finding of this study was that women with HG reported higher birth-related PTSS scores at 8 weeks postpartum than women who had experienced severe, mild or no nausea. Two years after giving birth, however, women with HG reported higher PTSS scores only when compared with women who had no nausea. The associations remained significant after additional adjustment for negative birth experience.

Our study was based on a longitudinal pregnancy cohort study with the focus on maternal mental health in relation to pregnancy, birth and the period up to 2 years following childbirth. To the best of our knowledge, no previous study has explored the association between the degree of nausea and childbirth-related PTSS. However, available studies show results along the lines of the results of the present study. A Danish register-based study of women with no psychiatric history (Meltzer-Brody et al. 2017) revealed that women with HG had a greater risk of developing acute stress (incidence rate ratio 1.32, 95% CI 1.33–2.55) up to 12 months following childbirth. In an American cross-sectional study utilising service usage data (Seng et al. 2001), women with PTSD were nearly four times more likely to have had HG than women without PTSD (aOR 3.9, 95% CI 2.0–7.4). In contrast to our study, the two studies noted above did not provide information about the specific trauma exposure. Two further studies based on an online survey (Christodoulou-Smith et al. 2011; Fejzo et al. 2009; Mullin et al. 2012) also showed that women with HG were more likely to experience postpartum PTSS. However, the definitions of PTSS were not adequately explained in these studies to render comparison possible.

In order to investigate, whether PTSS would change over time, we estimated the association between the degree of nausea during pregnancy and birth-related PTSS at two time points after birth. Indeed, our results revealed higher PTSS scores among women with HG at both time points compared to those with less severe nausea symptoms. The results also suggest that PTSS levels decrease over time.

As the PTSS score was related to childbirth, we wanted to explore whether differences in scores could be explained by the birth experience, since a negative birth experience has previously been shown to be the most important factor in the development of PTSS following childbirth (Garthus-Niegel et al. 2013). Thus, we estimated two multiple models, one adjusting and one not adjusting for negative birth experiences. Our results revealed that childbirth-related PTSS was strongly associated with negative birth experiences (Supplementary table). After additional adjustment for negative birth experiences in the adjusted model, there were still statistically significant differences in PTSS scores between the HG group and women with no, mild or severe nausea 8 weeks postpartum, suggesting that women with HG have a greater risk of PTSS regardless of their birth experience.

Although the differences in mean scores were small (2–4 points) and all mean scores were far below the scores that may indicate a full PTSD diagnosis (score > 34), we believe that the results may still be of clinical importance. Unfortunately, the nature of the data allowed us to investigate PTSS only 8 weeks and 2 years after birth, thus limiting the interpretation of development in the symptom burden. In future research, it would be interesting to explore whether stress levels among women who suffered from HG increase, when they consider a new pregnancy. When addressing the results of the present study, a few things are important to bear in mind. First, severe, unremitting nausea and vomiting is, for many women, a traumatic experience. The severity of nausea is comparable to that of receiving chemotherapy (Lacroix et al. 2000), and quality of life may decrease to a level comparable to that of women with breast cancer or myocardial infarction (Lacasse et al. 2008). In addition, feelings of isolation and lack of support may put a strain on a partner relationship (Heitmann et al. 2017; Lacasse et al. 2008; Mazzotta et al. 2000; Tan et al. 2017). Furthermore, thoughts of pregnancy termination (Heitmann et al. 2017; Poursharif et al. 2007) or suicidal ideation may arise (Poursharif et al. 2007). If a doctor hesitates to initiate treatment, this may worsen these negative emotions (Grooten et al. 2015; Raymond 2013). Current options of treatment are mainly restricted to symptom relief, such as anti-emetics, rehydration and supplementation of vitamins and nutrients (Grooten et al. 2015).

Our results indicate that HG may influence the whole pregnancy and birth experience in such a way that it increases the risk of PTSS following childbirth. Hence, women with HG may receive better care, if health care providers practice holistically, involving social and psychological care as well as physical interventions. In addition, improved health care and support may prevent women from resorting to either pregnancy termination due to anxiety about being pregnant (Mazzotta et al. 2000; Poursharif et al. 2007; Raymond 2013) or avoidance of future pregnancies (Heitmann et al. 2016; Poursharif et al. 2008).

### Strengths and limitations

A major strength of this study is its long-term, prospective data collection, which allowed us to assess PTSS scores 8 weeks and 2 years after childbirth. Self-selection bias may have been present; however, self-selection bias does not necessarily affect the estimated associations (Nilsen et al. 2009). No women were excluded due to other pregnancy complications, thereby reducing the risk of overestimating the association between the degree of nausea and birth-related PTSS. However, generalisability may be limited as only Norwegian-speaking women, mainly Caucasians, were included in the study. The associations may therefore differ in other ethnic groups.

We used instruments designed to measure psychological distress in population-based studies. Questionnaire studies often yield larger study populations compared with studies utilising clinical interviews, though questionnaires cannot be used for clinical diagnosis.

Subjective birth experience was measured retrospectively 2 months postpartum, but should ideally have been measured shortly after delivery. Additionally, the subjective birth experience and PTSS were assessed simultaneously, which may affect the causal direction; however, it seems reasonable to assume that the birth experience would influence the stress response rather than vice versa (Garthus-Niegel et al. 2013). No established and validated instrument such as the Perceptions of Labour and Delivery Scale (Bailham et al. 2004) was used in the present study to measure subjective birth experiences. Use of a more comprehensive and validated measurement instrument might provide further insights into the role of subjective birth experiences in the development of PTSS.

Regarding the definition of HG, the ABC study unfortunately does not include a validated score such as the Pregnancy Unique Quantification of Emesis (PUQE) (Birkeland et al. 2015; Koren et al. 2005). The data provided information about the degree of nausea, sick leave and hospitalisation. In Norway, during the study period, only women with severe metabolic disturbances were admitted to the hospital for HG, and no outpatient treatment was available. However, health care is free of charge in Norway, and all women in need of inpatient treatment are admitted regardless of socioeconomic status. Therefore, in order to restrict our HG group to those with the most severe symptoms, we defined HG as hospitalisation due to nausea. Hospitalisation for HG was assessed retrospectively in pregnancy week 32; however, recall bias is highly unlikely due to the relatively short interval (Vikanes et al. 2010b).

## Conclusion

Women with HG had higher PTSS scores following childbirth, compared with women with no nausea and those with less pronounced symptoms. After 2 years, women with HG still had higher PTSS scores compared with women with no nausea. These associations remained significant regardless of the subjective birth experience. Although the overall differences in PTSS scores were small, the results may still be of clinical relevance. Understanding the association between HG and PTSS and perinatal mental health in general may help to move us towards a better, possibly more holistic approach in caring for women with HG, both psychologically and physically.

## Electronic supplementary material

Supplementary Table (DOCX 18 kb)
