# Supplementary material for: The association between the degree of nausea in pregnancy and subsequent posttraumatic stress
Source: Arch Womens Ment Health. 2018 Sep 17;22(4):493–501. doi: 10.1007/s00737-018-0909-z (PMC6647437; doi:10.1007/s00737-018-0909-z)
Supplement: Supplementary file 1 — (DOCX 18 kb) [file 737_2018_909_MOESM1_ESM.docx]

**Supplementary Table** Unadjusted and adjusted^*^ mean PTSS scores, of all variables, with 95% CI and p-values at eight weeks (n=1,749) and two years (n= 1,193) after birth, Akershus Birth Cohort study, Norway, 2008-2012. Cases with at least one missing value on co-variates were excluded.

| Variable | Unadjusted | | Adjusted,  without Negative Birth Experience | | Adjusted,  with Negative Birth Experience | |
| --- | --- | --- | --- | --- | --- | --- |
|  | 8 weeks | 2 years | 8 weeks | 2 years | 8 weeks | 2 years |
| Nausea  No (ref)  Mild  Severe  HG | 19.6 (19.1; 20.1)  20.2 (19.8; 20.6)  20.7 (20.2; 21.1)  23.1 (20.6; 25.5) | 18.6 (18.1; 19.1)  19.1 (18.6; 19.5)  19.4 (18.9; 20.0)  21.5 (18.4; 24.6) | 19.8 (18.0; 21.6)  20.4 (18.6; 22.1)  20.6 (18.8; 22.4)  22.6 (19.8; 25.5) | 18.5 (16.4; 20.7)  19.1 (17.0; 21.1)  19.3 (17.2; 21.3)  21.3 (17.8; 24.8) | 19.8 (18.1; 21.6)  20.2 (18.5; 21.9)  20.4 (18.7; 22.1)  22.9 (20.2; 25.6) | 18.5 (16.4; 20.5)  18.9 (16.9; 20.9)  19.1 (17.1; 21.0)  21.5 (18.1; 25.0) |
| No vs. Mild  No vs. Severe  No vs. HG  Mild vs. Severe  Mild vs. HG  Severe vs. HG | 0.057  **0.002**  **0.006**  0.158  **0.023**  0.057 | 0.180  **0.035**  0.068  0.335  0.126  0.193 | 0.077  **0.018**  **0.019**  0.406  0.054  0.087 | 0.107  **0.047**  0.068  0.567  0.141  0.181 | 0.184  0.099  **0.008**  0.623  **0.019**  **0.027** | 0.177  0.115  **0.038**  0.702  0.075  0.092 |
| Negative Birth  Experience  No  Yes | 19.8 (19.6; 20.0)  28.3 (27.2; 29.4) | 18.8 (18.5; 19.1)  25.1 (23.8; 26.3) |  |  | 19.8 (18.1; 21.6)  27.2 (25.2; 29.3) | 18.5 (16.4; 20.5)  24.1 (21.7; 26.5) |
| No vs. yes | **<0.001** | **<0.001** |  |  | **<0.001** | **<0.001** |
| Previous PTSD  For 1 unit increase | 1.8 (1.4; 2.1) | 1.3 (0.9; 1.7) | 1.1 (0.8; 1.5) | 0.9 (0.5; 1.4) | 1.1 (0.7; 1.4) | 0.9 (0.5; 1.3) |
|  | **<0.001** | **<0.001** | **<0.001** | **<0.001** | **<0.001** | **<0.001** |
| Maternal age  For 1 year increase | -0.1 (-0.2;-0.1) | -0.1 (-0.2; -0.05) | 0.00 (-0.1; 0.05) | 0.01 (-0.1; 0.1) | -0.02 (-0.1; 0.04) | 0.001 (-0.1; 0.07) |
|  | **<0.001** | **0.001** | 0.897 | 0.796 | 0.559 | 0.981 |
| Parity  Primipara  Multipara | 21.0 (20.7; 21.4)  19.3 (19.0; 19.7) | 20.0 (19.6; 20.4)  18.1 (17.6; 18.5) | 19.8 (18.0; 21.6)  18.2 (16.2; 20.1) | 18.5 (16.4; 20.7)  16.6 (14.3; 18.9) | 19.8 (18.1; 21.6)  18.5 (16.6; 20.4) | 18.5 (16.4; 20.4)  16.8 (14.5; 19.0) |
| Primi vs. multi | **<0.001** | **<0.001** | **<0.001** | **<0.001** | **<0.001** | **<0.001** |
| Education  ≤12 years  >12 years | 20.8 (20.3; 21.2)  19.9 (19.6; 20.2) | 19.3 (18.8; 19.9)  18.9 (18.6; 19.3) | 19.8 (18.0; 21.6)  19.6 (17.8; 21.5) | 18.5 (16.4; 20.7)  18.5 (16.3; 20.7) | 19.8 (18.1; 21.6)  19.8 (18.0; 21.6) | 18.5 (16.4; 20.5)  18.5 (16.4; 20.7) |
| Low vs. high | **0.003** | 0.239 | 0.483 | 0.851 | 0.831 | 0.881 |
| Obstetric  complications  No  1 or more | 20.2 (19.9; 20.4)  20.4 (19.7; 21.0) | 19.0 (18.6; 19.3)  19.6 (18.9; 20.3) | 19.8 (18.0; 21.6)  19.6 (17.7; 21.5) | 18.5 (16.4; 20.7)  18.7 (16.5; 20.9) | 19.8 (18.1; 21.6)  19.6 (17.8; 21.4) | 18.5 (16.4; 20.5)  18.7 (16.5; 20.8) |
| No vs. 1 or more | 0.580 | 0.115 | 0.461 | 0.641 | 0.437 | 0.627 |
| Prenatal depression  No  Yes | 19.9 (19.6; 20.1)  25.0 (24.0; 26.1) | 18.8 (18.5; 19.1)  22.8 (21.6; 24.1) | 19.8 (18.0; 21.6)  22.5 (20.4; 24.6) | 18.5 (16.4; 20.7)  21.4 (18.9; 23.9) | 19.8 (18.1; 21.6)  22.1 (20.0; 24.1) | 18.5 (16.4; 20.5)  21.0 (18.6; 23.4) |
| No vs. Yes | **<0.001** | **<0.001** | **<0.001** | **<0.001** | **<0.001** | **<0.001** |
| Prenatal anxiety  No  Yes | 19.8 (19.5; 20.0)  25.2 (24.3; 26.1) | 18.9 (18.6; 19.2)  21.1 (20.0; 22.2) | 19.8 (18.0; 21.6)  23.2 (21.2; 25.2) | 18.5 (16.4; 20.7)  18.9 (16.6; 21.2) | 19.8 (18.1; 21.6)  23.0 (21.1; 24.9) | 18.5 (16.4; 20.5)  18.7 (16.4; 20.9) |
| No vs. Yes | **<0.001** | **<0.001** | **<0.001** | 0.541 | **<0.001** | 0.750 |
| Major negative  life events  None  1 or more | 19.5 (19.1; 19.8)  21.1 (20.7; 21.4) | 18.5 (18.1; 18.9)  19.8 (19.3; 20.2) | 19.8 (18.0; 21.6)  20.6 (18.8; 22.4) | 18.5 (16.4; 20.7)  19.3 (17.2; 21.4) | 19.8 (18.1; 21.6)  20.6 (18.9; 22.3) | 18.5 (16.4; 20.5)  19.2 (17.2; 21.2) |
| None vs. 1 or more | **<0.001** | **<0.001** | **0.002** | **0.009** | **0.002** | **0.010** |

*Adjusted for maternal age, parity, education, obstetric complications, previous PTSD, prenatal depression, prenatal anxiety, and major negative life events
